# Epstein-Barr virus associated diseases: Immune alterations and targeted approaches

**DOI:** 10.3389/fimmu.2026.1794394

**Published:** 2026-05-08

**Authors:** Shijia Cao, Jie Xiong, Jiayi Zhao, Wei-Li Zhao

**Affiliations:** Shanghai Institute of Hematology, State Key Laboratory of Medical Genomics, National Research Center for Translational Medicine at Shanghai, Ruijin Hospital Affiliated to Shanghai Jiao Tong University School of Medicine, Shanghai, China

**Keywords:** autoimmune, EBV - Epstein-Barr Virus, immune, targeted therapy, tumor microenvironment

## Abstract

Epstein-Barr virus (EBV) is a ubiquitous human γ-herpesvirus associated with a wide spectrum of diseases, ranging from autoimmune diseases to malignancies. Recognized as the first human oncogenic virus, multifaceted pathogenesis of EBV-associated diseases has been extensively explored. However, recent evidence suggests that EBV modulates immune cell function to influence the initiation and progression of associated diseases. Expressing a diverse repertoire of viral RNAs and proteins, EBV not only maintains latency with multiple immune evasion mechanisms, but also modulates host cell biology and sculpts a disease-specific immune microenvironment through dynamic interactions with various immune cells like T cells, B cells, macrophages, natural killer cells, and dendritic cells. Correspondingly, an increasing number of immunotherapies targeted T cells, B cells and virus itself have been developed for improved efficacy, including monoclonal antibodies, chimeric antigen receptor T cell (CAR-T) therapy, vaccines, and lytic induction strategies. In this review, regulatory roles of EBV in modulating the immune microenvironment in different context of disease and emerging therapeutic strategies targeted the underlying molecular mechanisms have been systematically discussed. Future research should aim to elucidate the precise molecular pathways involved, identify key therapeutic targets, characterize intra- and inter-tumor heterogeneity, and develop virus-directed strategies to fundamentally counteract EBV-induced immunopathogenic effects.

## 1 Introduction

**Figure 1:**
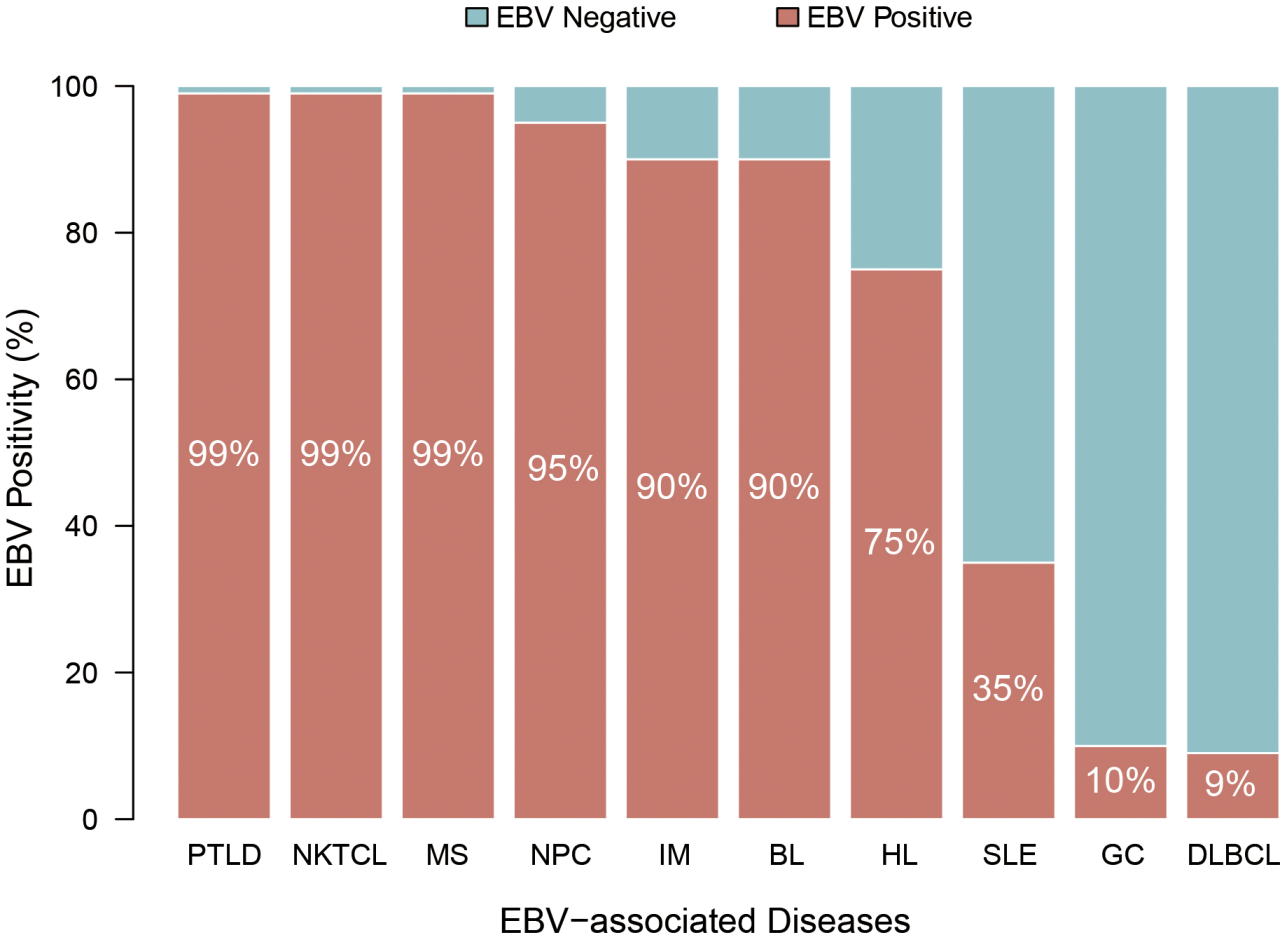
EBV positivity in associated diseases.

Epstein-Barr virus (EBV) is a ubiquitous human γ-herpesvirus that establishes lifelong infection in more than 90% of the global population (1). First discovered in tumor cells of Burkitt lymphoma (BL), EBV was subsequently recognized as the first human oncogenic virus (2). Beyond its oncogenic potential, EBV is implicated in a wide spectrum of diseases, including infectious mononucleosis (IM), multiple sclerosis (MS), and systemic lupus erythematosus (SLE), nasopharyngeal carcinoma (NPC), gastric cancer (GC), various lymphomas, and post-transplant lymphoproliferative disorder (PTLD) (**Figure 1**).

Primary EBV infection typically initiates in the epithelial cells of the oropharynx. Following viral replication and release from these epithelial cells, the virus accesses the lymphoid tissue of the tonsillar ring, where it infects B lymphocytes. EBV establishes latent infection within circulating memory B cells and undergoes lytic replication in a subset of plasma cells, generating infectious virions that subsequently infect epithelial cells at distal sites, such as the nasopharynx and stomach, or shed into saliva, thereby facilitating viral transmission (3). Recognized as the first oncogenic virus, EBV expresses multiple proteins that change biological behaviors of host cells in its latency, mediating cell malignant transformation. Latent membrane proteins LMP1 can activate the NF-κB and MAPK signaling pathways and modulate tumor suppressor genes such as *TP53*, thereby promoting cellular immortalization (4). In addition, LMP1 regulates epigenetic modifications by upregulating DNA methyltransferases (DNMTs), resulting in hypermethylation of host gene promoters (5), and subsequent disruption of cell cycle checkpoints, inhibition of DNA repair, and induction of genomic instability (6). Beyond signaling and epigenetic regulation, viral proteins also reprogram host cell metabolism. LMP1 enhances aerobic glycolysis to sustain anabolic metabolism and cellular proliferation (7), whereas Epstein-Barr nuclear antigen 2 (EBNA2) modulates the metabolic enzyme indoleamine 2,3-dioxygenase 1 (IDO1), thereby promoting *de novo* nicotinamide adenine dinucleotide (NAD) biosynthesis to support B cell transformation (8). In conclusion, EBV has been demonstrated to mediate cell malignant transformation by modulating tumor suppressor genes, epigenic modifications and cell metabolisms of host cells.

Except for interfering biological behaviors of host cells, growing evidence suggests that EBV modulates immune cell function and contributes to the immune pathogenesis of multiple diseases. In this review, we focus on the regulatory roles of EBV in modulating the immune microenvironment in different context of diseases, and summarize the underlying molecular mechanisms and emerging therapeutic strategies.

## 2 EBV mediates immune evasion to maintain latency

EBV establishes latent infection in host cells through four distinct latency programs, classified according to the pattern of viral gene expression. Latency 0, commonly observed in healthy carriers, is characterized by the expression of only Epstein-Barr virus encoded RNAs (EBERs) and BamHI-A rightward transcripts (BARTs). Latency I, typically detected in GC and BL, involves expression of EBNA1 in addition to non-coding RNAs. Latency II, observed in NPC, GC, Hodgkin lymphoma (HL), NK/T-cell lymphoma (NKTCL), diffuse large B-cell lymphoma (DLBCL), MS, and SLE, is defined by expression of EBNA1 together with LMP1 and LMP2. Latency III represents the most transcriptionally active program, with expression of nearly all latent proteins and non-coding RNAs, and is typically found in DLBCL and PTLD (2). The heterogeneity of these latency-associated gene expression profiles underscores the diverse pathogenic strategies employed by EBV in both malignant and non-malignant diseases.

**Figure 2:**
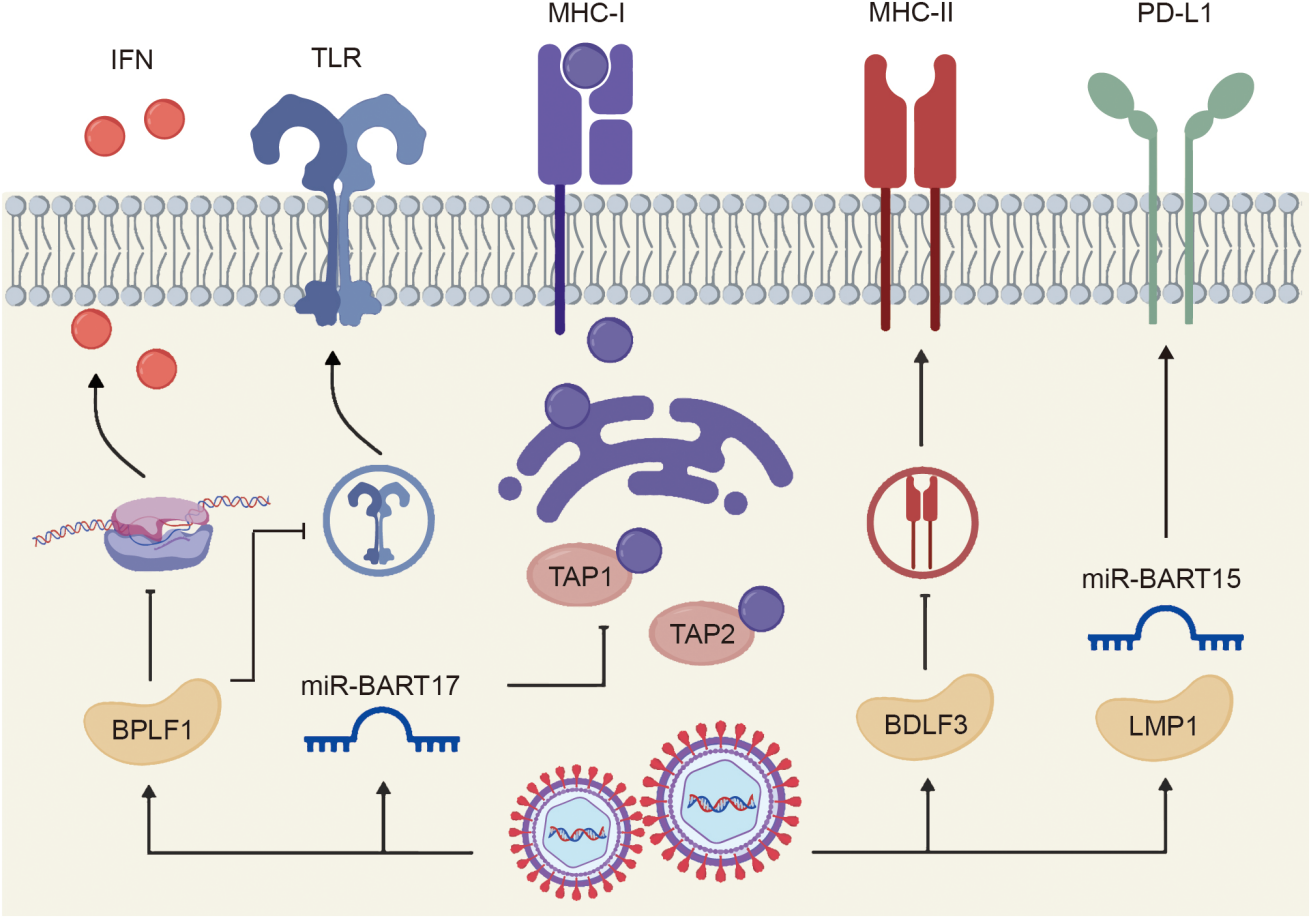
EBV mediates immune evasion to maintain latency.

Primary EBV infection elicits both innate and adaptive immune responses. However, the virus employs multiple immune evasion strategies to establish and maintain latency within host cells. Innate immune recognition of EBV is primarily mediated through pattern recognition receptors (PRRs) and interferon (IFN) signaling pathways. To counteract these mechanisms, EBV-encoded miR-BARTs (9) and the viral protein BamHI P leftward reading frame 1 (BPLF1) (10) downregulate Toll-like receptor (TLR) signaling and suppress IFN production, thereby attenuating antiviral responses. Adaptive immune evasion is achieved through interference with the function of antigen presentation cells and cytotoxic lymphocytes. EBV-derived microRNAs, such as miR-BART17, inhibit the transporter associated with antigen processing 1 and 2 (TAP1 and TAP2), preventing peptide transport into the endoplasmic reticulum (ER) and subsequent loading onto MHC class I (MHC-I) molecules. In addition, the viral glycoprotein BamHI-D leftward reading frame 3 (BDLF3) disrupts the presentation of MHC-I and MHC-II molecules, thereby limiting T cell activation. Moreover, LMP1 and miR-BART5 activate the STAT3 signaling pathway, inducing PD-L1 expression and promoting apoptosis or exhaustion of cytotoxic lymphocytes (11). With those strategies inhibiting both innate and adaptive immune responses, EBV can maintain lifelong latency within host cells (**Figure 2**).

## 3 EBV regulates the immune microenvironment contributing to pathogenesis

**Figure 3:**
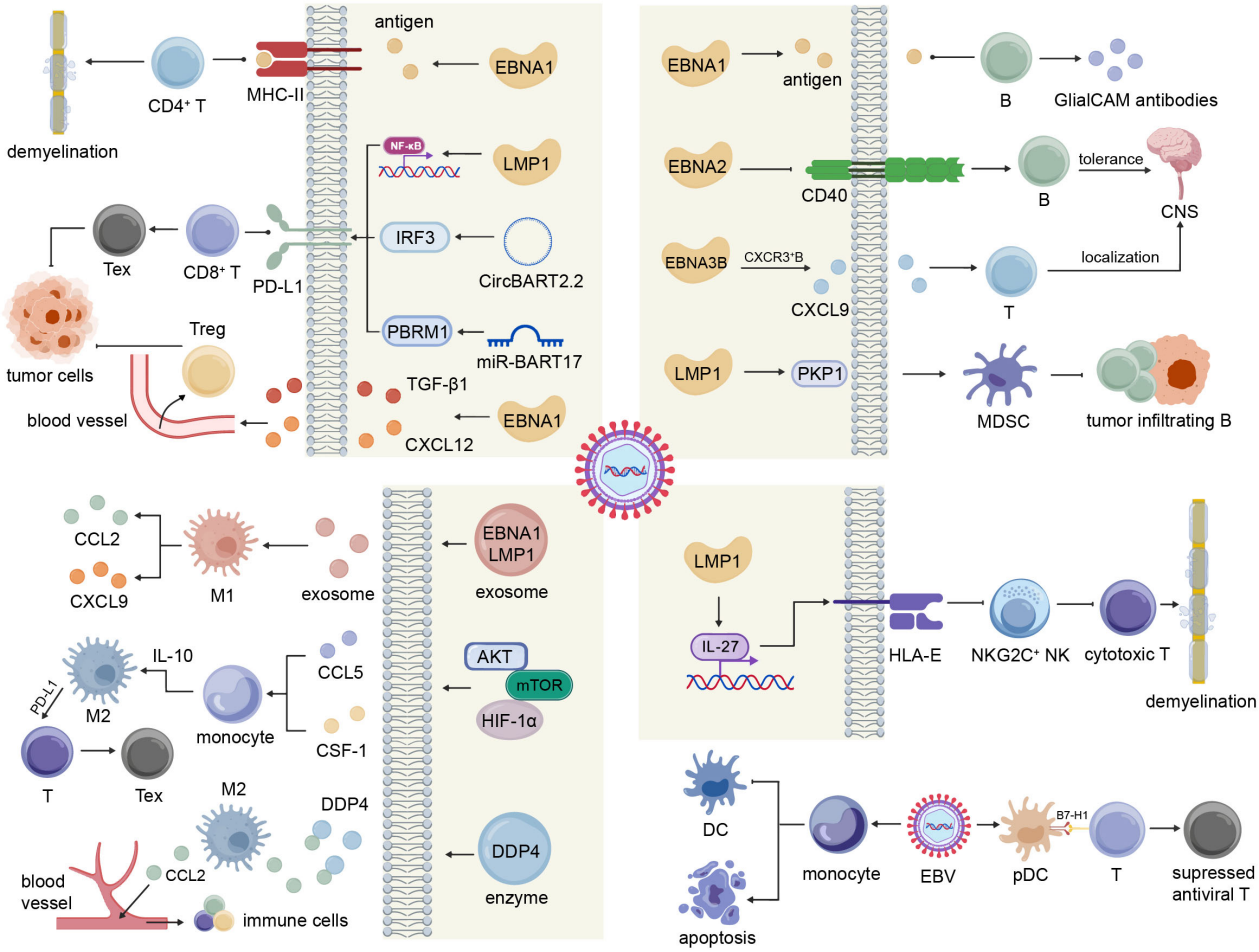
EBV modulates T cells, B cells, macrophages, NK cells and dendritic cells.

EBV expresses a diverse repertoire of viral RNAs and proteins that not only maintain latency in epithelial and B cells but also modulate host cell biology and sculpt a disease-specific immune microenvironment through dynamic interactions with various immune cells. In autoimmune diseases, immune cells within and surrounding lesions often exhibit hyperactivation and autoreactivity, contributing to tissue damage and autoimmune pathology. By contrast, in malignancies, EBV drives cellular transformation and facilitates the establishment of an immunosuppressive tumor microenvironment (TME), in which infiltrating immune cells frequently acquire tumor-tolerogenic phenotypes. This section discusses the multifaceted interactions between EBV and immune cells and how these interactions influence disease initiation and progression (**Figure 3**).

### 3.1 T cells

CD4^+^ T cells within the central nervous system (CNS) can recognize EBNA1 and cross-react with myelin antigens through molecular mimicry in MS. These EBNA1-specific CD4^+^ T cells display heightened activation frequency and responsiveness, suggesting a pivotal role in MS pathogenesis (12). Moreover, histopathological analyses have confirmed that CD8^+^ T cells migrate into and infiltrate the CNS, localizing to areas of microglial proliferation and myelin sheath damage. These infiltrating cytotoxic T cells secrete effector molecules that, together with phagocytic myeloid cells, contribute to demyelination of the CNS (13). In SLE, increased differentiation of T helper 17 (Th17) cells is associated with EBV infection. EBV-induced IFN-γ signaling activates the STAT3 pathway and downregulates the transcription factor cellular musculoaponeurotic fibrosarcoma oncogene homolog (c-Maf), thereby promoting Th17 differentiation. The resulting Th17 cells release cytokines such as interleukin-17 (IL-17), which perpetuate a chronically inflamed immune microenvironment (14).

In tumors, CD8^+^ T cells infiltrated in the TME often exhibits a high expression of exhaustion markers such as PD-1, LAG3, and TIM3, indicative of impaired cytotoxic function (15). EBV has been shown to induce CD8^+^ T cell exhaustion through multiple mechanisms. First, EBV interferes the expression of MHC on tumor-antigen presenting cells and tumor cells to disturb the recognition of CD8^+^ T cells (16). In EBV-associated gastric cancer, EBV upregulates MHC-II expression in B cells, dendritic cells (DCs), and macrophages (17), whereas in DLBCL and HL, MHC-I downregulation mediated by EBV occurs in more than 70% cases (18). Second, both EBV-encoded proteins and RNAs can enhance PD-L1 expression on tumor cells, enabling interaction with PD-1 on T cells to suppress their activation and cytotoxic function. For example, LMP1 upregulates PD-L1 through the NF-κB/MAPK/AP1 signaling pathway (19). CircBART2.2 binds to the helicase domain of RIG-1, thereby activating the transcription factors IRF3 and NF-κB to induce PD-L1 expression (20). Moreover, miR-BART17 targets the transcriptional inhibitor PBRM1, promoting its degradation and relieving its repressive effect on PD-L1 transcription (21). Besides, Treg cell infiltration represents another major immunosuppressive feature of the TME. In NPC, multiple studies have demonstrated that EBV recruits and promotes the differentiation of Treg cells. For example, EBNA1 has been shown to promote tumor cells to secrete TGF-β1, thereby recruiting peripheral Treg cells through the SMAD3-PI3K-AKT-c-JUN-CXCL12-CXCR4 signaling axis. EBNA1 also downregulates miR-200a to further enhance CXCL12 expression, cooperatively attracting CXCR4^+^ Tregs (22). Moreover, EBV upregulates NFKB2 to induce CD70 expression, which interacts with CD27 to drive naïve CD4^+^ T cell differentiation into Tregs and enhances their immunosuppressive activity by initiating lipid metabolic reprogramming (23).

### 3.2 B cells

In autoimmune diseases, B cells contribute directly to pathogenesis and disease progression through the production of autoreactive antibodies. In MS, clonally expanded B cells in the cerebrospinal fluid (CSF) produce oligoclonal immunoglobulins that specifically recognize EBNA1 and cross-react with the glial cell adhesion molecule (GlialCAM), thereby mediating immune-driven demyelination (24). The generation of aberrant antibodies in MS is associated with multiple B cell dysfunctions, including excessive pro-inflammatory cytokine secretion, impaired regulatory activity, and the formation of tertiary lymphoid structures (TLSs) within the CNS (25). Elevated expression of EBV lytic genes has been detected in B cells during active phases of MS, suggesting that viral reactivation may drive B cell dysregulation (26). Moreover, EBNA2 1.2 allele has been shown to downregulate CD40, a key molecule involved in maintaining B cell CNS tolerance within the thymus, further implicating EBV genes in the disruption of normal B cell immune regulation in MS (27). Similarly in SLE, LMP2A has been shown to enable autoreactive anti-Smith B cells to bypass tolerance checkpoints, thereby promoting autoantibody production (28).

In MS, B cells residing within the CNS can recruit T cells and thereby exacerbate demyelination except by aberrant antigen recognition and autoantibody production. Studies have shown that EBV infection increases the frequency of T-bet^+^CXCR3^+^ B cells in the brain, spleen, and peripheral blood. These CXCR3-dependent B cells preferentially localize beneath the meninges, where they contribute to local immune activation. Furthermore, the EBV latent protein EBNA3B induces these B cells to secrete the chemokine CXCL9, which recruits activated T cells that act in concert with other chemokines, including CCL3, CCL4, and CCL5 (29).

The role of B cells in malignant diseases is highly heterogeneous. In hematologic malignancies, EBV directly drives the malignant transformation of B cells, contributing to the development of B cell lymphomas such as DLBCL. In contrast, in solid tumors such as NPC, B cell infiltration is often correlated with improved clinical outcomes. Studies have shown that in NPC, tumor-infiltrating B cells within TLSs can function as antigen-presenting cells to activate T cells, recruit other immune populations through cytokine secretion, and produce tumor-specific antibodies that mediate anti-tumor immunity (30). However, EBV-infected NPC cells express LMP1 and PKP1, which recruit myeloid-derived suppressor cells (MDSCs) to eliminate B cells, thereby facilitating immune evasion (31).

### 3.3 Macrophages

Macrophages exhibit two major functional phenotypes: the pro-inflammatory M1 and the immunosuppressive M2 subtype. In autoimmune diseases, macrophages release pro-inflammatory cytokines that contribute to tissue damage and disease progression. During the active phase of MS, exosomes containing EBNA1 and LMP1 have been shown to stimulate macrophages to secrete CXCL9 and CCL2, indicating that EBV-encoded factors promote macrophage-mediated neuroinflammation (32). Moreover, EBV infection has been reported to diminish macrophage interactions with memory T cells while enhancing their contact with glial cells, further suggesting that EBV contributes to MS pathogenesis by altering the functional and spatial dynamics of macrophages (33).

Tumor-associated macrophages (TAMs) predominantly display an M2 phenotype and play a central role in establishing an immunosuppressive microenvironment. EBV promotes infected NPC cells to secrete CCL5 and CSF-1 via the AKT/mTOR/HIF-1α signaling pathway, which recruits peripheral monocytes and induces autocrine IL-10 production, thereby driving their polarization into CD163^+^ M2 macrophages (34). TAMs modulate the TME as a double-edged sword, as they suppress T cell-mediated anti-tumor immunity through multiple immune checkpoint interactions, including CD80/CD86-CTLA4, PD-L1/PD-L2-PD-1, and LGALS9-HAVCR2, while TAM-derived chemokines such as CCL2 and CXCL2 can also recruit peripheral immune cells. However, in NKTCL, EBV^+^ malignant natural killer cells express DPP4, which degrades these chemokines and synergistically promotes immune evasion (35). Beyond immune modulation, TAMs also promote epithelial-mesenchymal transition (EMT) and angiogenesis, as well as CCL18-mediated NF-κB activation, thereby facilitating tumor growth and metastasis in NPC (36).

### 3.4 Natural killer cells

Studies have demonstrated that the frequency of NKG2C^+^ NK cells is significantly lower in patients with MS than in healthy individuals with EBV infection. NKG2C^+^ NK cells have been identified as key regulators that control GlialCAM-specific cytotoxic T cells, whereas in MS, EBV infection promotes IL-27 signaling, which upregulates human leukocyte antigen-E (HLA-E) expression and thereby suppresses NKG2C^+^ NK cell activity. Comparative genomic analyses of EBV strains from patients with MS and healthy individuals have revealed that nearly all patients with MS harbor two specific mutations in the LMP1 gene, suggesting that aberrant HLA-E overexpression may be mediated by LMP1 variants (37).

### 3.5 Dendritic cells

DCs are pivotal antigen-presenting cells that initiate and coordinate antiviral immune responses. Studies have shown that the addition of DCs to co-cultures of EBV^+^ B cells and T cells inhibits clonal B cell expansion, underscoring the importance of DC-mediated cross-presentation in T cell-driven antiviral immunity (38). Similarly, DCs loaded with LMP2A can stimulate the proliferation of antigen-specific CD4^+^ and CD8^+^ T cells (39). However, in EBV-associated malignancies, DC function is frequently impaired through multiple mechanisms. EBV infection of monocytes has been shown to inhibit their differentiation into DCs and to induce aberrant responses to GM-CSF and IL-4 (40). Moreover, EBV blocks the maturation of plasmacytoid DCs (pDCs) and upregulates T cell inhibitory ligands such as B7-H1 and ICOS-L, thereby suppressing antiviral T cell responses (41).

### 3.6 Convergent immunomodulatory mechanisms across EBV-associated diseases

Despite the phenotypic diversity of EBV-associated diseases, our analysis reveals that EBV has evolved shared immunomodulatory strategies targeting critical nodes of immune surveillance. First, a unifying feature is the convergent activation of NF-κB and STAT3 signaling as central hubs for immune regulation. In SLE, EBV-driven STAT3 activation promotes Th17 cell differentiation, thereby fostering an inflammatory microenvironment (14). By contrast, in malignancies, EBV converges on NF-κB activation through multiple routes to upregulate PD-L1 and establish an immunosuppressive TME, including LMP1, circBART2.2 and miR-BART17 (19, 20; 21).

Second, EBV orchestrates a paradoxical attract-and-suppress strategy through systematic hijacking of chemokine networks. In MS, EBV recruits cytotoxic T cells to sites of demyelination via CXCL9 to mediate autoimmune pathology (29). Conversely, in NPC, EBV recruits immunosuppressive Treg cells and M2 macrophages via the CXCL12-CXCR4 and CCL5-CSF-1 axes to establish tumor immune tolerance (22). Furthermore, in NKTCL, EBV degrades CCL2 and CXCL2 to evade anti-tumor immunity (35).

Third, EBV implements layered disruption of MHC expression to control immune recognition. In MS, EBV upregulates HLA-E to suppress NKG2C^+^ NK cells, thereby removing a critical checkpoint for controlling GlialCAM-specific autoreactivity (37). Conversely, in malignancies, EBV downregulates MHC-I in DLBCL and HL, and upregulates MHC-II in GC to mediate CD8^+^ T cell exhaustion, thereby suppressing anti-tumor immunity (16–18).

In summary, EBV orchestrates a pro-inflammatory immune microenvironment by activating and modulating CD4^+^ T cells, Th17 cells, B cells, and macrophages, while suppressing NK cell activity, thereby driving the onset and progression of autoimmune diseases. In contrast, within EBV-associated malignancies, the virus promotes CD8^+^ T cell exhaustion, Treg cell infiltration, B cell transformation and depletion, DC dysfunction, and macrophage M2 polarization, collectively facilitating tumor immune evasion and invasion. These findings highlight that the pathogenesis of EBV-associated diseases arises from a complex interplay between host genetic susceptibility and environmental factors, with EBV-induced reprogramming of the immune microenvironment representing a critical determinant of disease development and progression.

## 4 Targeted approaches of EBV-associated diseases

As our understanding of EBV-mediated immune alterations continues to expand, an increasing number of EBV-associated diseases are achieving improved outcomes with the advent of immunotherapies. This section summarizes immune-based therapeutic strategies and molecular targets for both autoimmune and malignancy-associated diseases, providing an overview of the current landscape of EBV-directed therapeutic approaches (**Table 1**).

**Table 1:** Targeted approaches of EBV-associated diseases.

Target cells	Target molecules	Approaches	Diseases	Evidence level	EBV status
T cells	PD-1	Toripalimab (42)	*NPC	*SOC (FDA approved)	EBV-independent
		Nivolumab (43); Pembrolizumab (44)	*HL	SOC (FDA approved)	EBV-independent
	CTLA4	Ipilimumab (45)	NPC	Phase II clinical trials (NCT03097939)	Low plasma EBV-DNA titre (<7800 IU/ml) trend to better response
B cells	CD20	Ocrelizumab (46); Ofatumumab (47); Ublituximab (48)	*MS	SOC (FDA approved)	EBV-independent
		Rituximab (49)	MS; *SLE	Guideline-supported but off-label	EBV-independent
			*NHL	SOC (FDA approved)	EBV-independent
	CD19	Axicabtagene ciloleucel (50); Tisagenlecleucel (51); Lisocabtagene maraleucel (52)	*DLBCL; *FL	SOC (FDA approved)	EBV-independent
	BTK	Fenebrutinib (53)	MS	Phase II clinical trials (NCT05119569)	EBV-independent
		Ibrutinib (54); Acalabrutinib (55); Zanubrutinib (56)	*MCL	SOC (FDA approved)	EBV-independent
	BAFF	Belimumab (57)	SLE	SOC (FDA approved)	EBV-independent
EBV	LMP1	LMP1-directed CAR-T (58)	NPC	Preclinical	EBV^+^
	gH/gL, gp42, gp220	mRNA-1189 vaccine	EBV prevention	Phase I clinical trials (NCT05164094)	EBV^-^
	gp350	gp350-Ferritin nanoparticle vaccine (59)	EBV prevention	Phase I clinical trials (NCT04645147)	EBV^-^
	EBNA2, LMP2	MVA-EL vaccine (60)	NPC	Phase II clinical trials (NCT01094405)	EBV^+^
	Multiple EBV antigens	WGc-043 mRNA vaccine (61)	NPC	Phase I clinical trials (NCT05714748)	EBV^+^
	B-LCL lysates	Ksd-101 DC vaccine (62)	EBV-associated hematologic neoplasms	Phase I Clinical Study (NCT05635591)	EBV^+^
	BZLF1	mTZ3-LNP (63)	EBV-associated epithelial cancers	Preclinical	EBV^+^

*SOC (FDA approved), standard-of-care (U.S food and drug administration approved); *NPC, nasopharyngeal carcinoma; *HL, Hodgkin lymphoma; *MS, multiple sclerosis; *SLE, systemic lupus erythematosus; *NHL, non-Hodgkin lymphoma; *DLBCL, diffuse large B cell lymphoma; *FL, follicular lymphoma; *MCL, mantle cell lymphoma.

### 4.1 T cell-targeted approaches

Standard treatments for EBV-associated malignancies traditionally include chemotherapy and radiotherapy. However, with growing insights into the tumor immune microenvironment, immune-targeted therapies have emerged as promising therapeutic alternatives. Given the EBV-mediated upregulation of PD-1/PD-L1 and the resultant T cell exhaustion, anti-PD-1 monoclonal antibodies have become widely adopted immune checkpoint inhibitors, among which toripalimab has demonstrated significant efficacy in NPC (42), as well as nivolumab (43) and pembrolizumab (44) in HL. CTLA4 represents another inhibitory immune checkpoint. Its monoclonal antibody ipilimumab has been proved by phase II clinical trial to be of clinical benefit in recurrent/metastatic NPC, with low plasma EBV-DNA titre trending to better response (45).

### 4.2 B cell-targeted approaches

Given the central role of autoreactive oligoclonal antibodies in the pathogenesis and diagnosis of MS, B cell-depleting therapies have become a mainstay of treatment. Among these, anti-CD20 monoclonal antibodies represent the most extensively developed, selective, and widely used approach, including ocrelizumab (46), ofatumumab (47), and ublituximab (48). Another class of B cell-targeted therapies focuses on Bruton’s tyrosine kinase (BTK), a signaling molecule that promotes the proliferation and survival of B cell-derived malignancies and has long been a therapeutic target in mantle cell lymphoma (54–56) and chronic lymphocytic leukemia (64). BTK inhibitors (BTKi) previously evaluated for MS included evobrutinib (65) and tolebrutinib (66), while neither demonstrated superior efficacy compared with the first-line immunomodulator teriflunomide (67). Notably, fenebrutinib has shown clinical benefit in a phase II clinical trial and may become the first approved BTKi for MS (53).

Given the analogous pathogenic role of B cells in SLE, the anti-CD20 monoclonal antibody rituximab has also been employed in SLE treatment, while it is guideline supported but off-label. Another targeted approach focuses on the B-cell activating factor (BAFF), which is overexpressed in SLE and promotes the survival and expansion of autoreactive B cells (68). The anti-BAFF monoclonal antibody belimumab blocks BAFF-mediated stimulation of B cells, thereby reducing their activation and prolongation of survival (69).

In non-Hodgkin lymphoma (NHL), the anti-CD20 monoclonal antibody rituximab remains the first-line therapeutic option (49). Another promising immunotherapeutic strategy is chimeric antigen receptor T cell (CAR-T) therapy, which has shown remarkable efficacy in hematologic malignancies, with CD19 serving as the most established target. Axicabtagene ciloleucel (axi-cel, Yescarta) was the first CAR-T therapy approved for diffuse large B cell lymphoma (DLBCL) and follicular lymphoma (FL) (50), whereas tisagenlecleucel (Kymriah) (51) and lisocabtagene maraleucel (liso-cel, Breyanzi) (52) have demonstrated efficacy in these indications.

### 4.3 EBV-targeted approaches

Given the pivotal role of EBV infection and viral gene expression in the pathogenesis and progression of malignancies, therapeutic strategies targeting EBV-associated molecules are actively being explored. To prevent EBV infection at its source, multiple prophylactic vaccines have been developed that primarily target EBV envelope glycoproteins mediating viral entry; neutralizing antibodies against these entry factors have been shown to effectively block EBV infection in experimental systems. Notably, the mRNA-1189 vaccine, which targets gH/gL, gp42 and gp350, as well as a gp350-ferritin nanoparticle vaccine, have entered phase I clinical trials (59). However, no vaccines have yet been approved for EBV prevention.

Studies have confirmed that EBV^+^ tumor cells express multiple viral latent antigens that contribute to immune escape; accordingly, strategies are being investigated to elicit antigen-specific CD4^+^ and CD8^+^ T cell responses against these malignancies, including CAR-T therapies and therapeutic vaccines. As EBV establishes latent infection in associated malignancies through latency II or latency III programs, both of which express EBNA1, LMP1, and LMP2, multiple studies have sought to target these antigens to eliminate tumor cells in NPC and hematologic neoplasms. LMP1-directed CAR-T cells have been shown to mediate potent anti-tumor activity in EBV^+^ NPC cells in preclinical models (58). However, LMP1 expression demonstrates considerable heterogeneity in EBV^+^ NPC, as immunohistochemical analyses indicate that LMP1 expression is significantly lower in advanced-stage NPC, suggesting that LMP1-directed CAR-T cells may be of limited clinical benefit for these patients (70). Furthermore, NPC cells with high LMP1 expression contribute to an immunosuppressive tumor microenvironment through multiple signaling pathways like NF-κB/MAPK/AP1 (71), indicating that LMP1-directed CAR-T cells must overcome the immune-evasive functions of their target antigen. Although peripheral blood mononuclear cells (PBMCs) from patients with EBV^+^ NPC do not express detectable LMP1, indicating a favorable safety profile for this approach (58), the clinical efficacy of the LMP1-directed CAR-T cell therapy in NPC remains to be validated in large-scale trials.

For therapeutic vaccines, a phase I clinical trial demonstrated that the MVA-EL vaccine, encoding a fusion protein composed of the carboxyl terminus of EBNA1 and full-length LMP2, effectively induced the proliferation of EBNA1- and LMP2-specific CD4^+^ and CD8^+^ T cells in EBV^+^ NPC (60). EBNA1 and LMP2, which are also expressed in latency II and III programs, appear more stably expressed than LMP1 in EBV^+^ NPC. Nevertheless, this trial identified several patients who did not derive clinical benefit, suggesting that potential antigen loss or immune escape mechanisms mediated by EBNA1 and LMP2 remain to be elucidated. Accordingly, additional clinical trials are required to validate the efficacy and safety of this approach. Other studies have developed vaccines targeting multiple EBV antigens, including the WGc-043 mRNA vaccine for NPC (61) and the Ksd-101 DC vaccine for EBV-associated hematologic neoplasms (62), both of which are currently in phase I clinical trials. The WGc-043 vaccine employs an artificial intelligence-designed, broad-spectrum antigen cocktail from latency II antigens to overcome single-antigen heterogeneity, whereas Ksd-101 utilizes autologous DCs loaded with lysates from whole EBV-transformed B lymphoblastoid cell lines to present a diverse array of antigens expressed in latency III, theoretically reducing the risk of immune escape through antigen loss. However, the safety profiles of these multi-antigen approaches require careful evaluation, as broad immune activation may increase the risk of autoimmunity or excessive inflammation.

Given the critical role of viral latency in EBV-associated malignancies, induction of the lytic cycle has also been explored as an anti-tumor strategy. EBV predominantly maintains latency II in NPC, with lytic genes such as *BZLF1* suppressed through epigenetic silencing. Recent studies have investigated mTZ3-LNP, a lipid nanoparticle-encapsulated mRNA encoding a BZLF1-specific TALE-transcriptional activator, which specifically activates BZLF1 expression and thereby induces tumor cell lysis (63). Although switching EBV^+^ cells from latent to lytic cycle can induce growth arrest, promote apoptosis, and cause lytic rupture, the potential risks of abortive lytic cycle and spontaneous lytic infection must be considered, as these may paradoxically drive tumor progression (72, 73). Furthermore, latency and lytic heterogeneity may result in variable clinical outcomes (74); accordingly, lytic induction strategies remain to be validated in large-scale clinical trials.

In conclusion, EBV-mediated immune alterations provide the rationale for therapeutic strategies in EBV-associated diseases. Although substantial progress has been made in understanding the complex interactions between EBV and the immune microenvironment, the underlying molecular mechanisms remain incompletely defined, and clinically effective EBV-targeted therapies are still under investigation. Future research aiming to elucidate the precise molecular pathways involved, identify key therapeutic targets, characterize intra- and inter-tumor heterogeneity, and develop virus-directed strategies to fundamentally counteract EBV-induced immunopathogenic effects are anticipated.

## References

[1] Macsween KF Crawford DH . Epstein-Barr virus—recent advances. Lancet Infect Dis. (2003) 3:131–40. doi: 10.1016/S1473-3099(03)00543-7. PMID: 12614729

[2] Damania B Kenney SC Raab-Traub N . Epstein-Barr virus (EBV): biology and clinical disease. Cell. (2022) 185:3652–70. doi: 10.1016/j.cell.2022.08.026. PMID: 36113467 PMC9529843

[3] Farrell PJ . Epstein-Barr virus and cancer. Annu Rev Pathol. (2019) 14:29–53. doi: 10.1146/annurev-pathmechdis-012418-013023. PMID: 30125149

[4] Liu H Tang L Li Y Xie W Zhang L Tang H . Nasopharyngeal carcinoma: current views on the tumor microenvironment’s impact on drug resistance and clinical outcomes. Mol Cancer. (2024) 23:20. doi: 10.1186/s12943-023-01928-2. PMID: 38254110 PMC10802008

[5] Chow L-Y Chung D-S Tao L Chan KF Tung SY Ngan RKC . Epigenomic landscape study reveals molecular subtypes and EBV-associated regulatory epigenome reprogramming in nasopharyngeal carcinoma. eBioMedicine. (2022) 86:104357. doi: 10.1016/j.ebiom.2022.104357. PMID: 36371985 PMC9663866

[6] Leong MML Cheung AKL Dai W Tsao SW Tsang CM Dawson CW . EBV infection is associated with histone bivalent switch modifications in squamous epithelial cells. Proc Natl Acad Sci USA. (2019) 116:14144–53. doi: 10.1073/pnas.1821752116. PMID: 31235597 PMC6628793

[7] Yang T You C Meng S Lai Z Ai W Zhang J . EBV infection and its regulated metabolic reprogramming in nasopharyngeal tumorigenesis. Front Cell Infect Microbiol. (2022) 12:935205. doi: 10.3389/fcimb.2022.935205. PMID: 35846746 PMC9283984

[8] Müller-Durovic B Jäger J Engelmann C Schuhmachers P Altermatt S Schlup Y . A metabolic dependency of EBV can be targeted to hinder B cell transformation. Science. (2024) 385:eadk4898. doi: 10.1126/science.adk4898. PMID: 38781354

[9] Bouvet M Voigt S Tagawa T Albanese M Chen Y-F Chen Y . Multiple viral microRNAs regulate interferon release and signaling early during infection with Epstein-Barr virus. mBio. (2021) 12:e03440-20. doi: 10.1128/mBio.03440-20. PMID: 33785626 PMC8092300

[10] Lui W-Y Bharti A Wong N-H Jangra S Botelho MG Yuen K-S . Suppression of cGAS- and RIG-I-mediated innate immune signaling by Epstein-Barr virus deubiquitinase BPLF1. PloS Pathog. (2023) 19:e1011186. doi: 10.1371/journal.ppat.1011186. PMID: 36802409 PMC9983872

[11] Yuan L Zhong L Krummenacher C Zhao Q Zhang X . Epstein–Barr virus-mediated immune evasion in tumor promotion. Trends Immunol. (2025) 46:386–402. doi: 10.1016/j.it.2025.03.007. PMID: 40240193

[12] Behrens M Comabella M Lünemann JD . EBV-specific T-cell immunity: relevance for multiple sclerosis. Front Immunol. (2024) 15:1509927. doi: 10.3389/fimmu.2024.1509927. PMID: 39776919 PMC11703957

[13] Allanach JR Fettig NM Hardman BK Rosen AR Fan V Chung C . Epstein-Barr virus infection promotes T cell dysregulation in a humanized mouse model of multiple sclerosis. Sci Adv. (2025) 11:eadu5110. doi: 10.1126/sciadv.adu5110. PMID: 40043135 PMC11881922

[14] Zhang Y Wang J Fang Y Liang W Lei L Wang J . IFN-α affects Th17/Treg cell balance through c-Maf and associated with the progression of EBV- SLE. Mol Immunol. (2024) 171:22–35. doi: 10.1016/j.molimm.2024.05.003. PMID: 38749236

[15] Nguyen LT Ohashi PS . Clinical blockade of PD1 and LAG3 — potential mechanisms of action. Nat Rev Immunol. (2015) 15:45–56. doi: 10.1038/nri3790. PMID: 25534622

[16] Choi I-K Wang Z Ke Q Hong M Paul DW Fernandes SM . Mechanism of EBV inducing anti-tumour immunity and its therapeutic use. Nature. (2021) 590:157–62. doi: 10.1038/s41586-020-03075-w. PMID: 33361812 PMC7864874

[17] Qiu M-Z Wang C Wu Z Zhao Q Zhao Z Huang C-Y . Dynamic single-cell mapping unveils Epstein–Barr virus-imprinted T-cell exhaustion and on-treatment response. Signal Transd. Targ. Ther. (2023) 8:1–13. doi: 10.1038/s41392-023-01622-1. PMID: 37735150 PMC10514267

[18] Roemer MGM Redd RA Cader FZ Pak CJ Abdelrahman S Ouyang J . Major histocompatibility complex class II and programmed death ligand 1 expression predict outcome after programmed death 1 blockade in classic Hodgkin lymphoma. J Clin Oncol. (2018) 36:942–50. doi: 10.1200/JCO.2017.77.3994. PMID: 29394125 PMC5877802

[19] Bi X Wang H Zhang W Wang J Liu W Xia Z . PD-L1 is upregulated by EBV-driven LMP1 through NF-κB pathway and correlates with poor prognosis in natural killer/T-cell lymphoma. J Hematol Oncol. (2016) 9:109. doi: 10.1186/s13045-016-0341-7. PMID: 27737703 PMC5064887

[20] Ge J Wang J Xiong F Jiang X Zhu K Wang Y . Epstein–Barr virus–encoded circular RNA CircBART2.2 promotes immune escape of nasopharyngeal carcinoma by regulating PD-L1. Cancer Res. (2021) 81:5074–88. doi: 10.1158/0008-5472.CAN-20-4321. PMID: 34321242 PMC8974435

[21] Wang J Ge J Wang Y Xiong F Guo J Jiang X . EBV miRNAs BART11 and BART17-3p promote immune escape through the enhancer-mediated transcription of PD-L1. Nat Commun. (2022) 13:866. doi: 10.1038/s41467-022-28479-2. PMID: 35165282 PMC8844414

[22] Huo S Luo Y Deng R Liu X Wang J Wang L . EBV-EBNA1 constructs an immunosuppressive microenvironment for nasopharyngeal carcinoma by promoting the chemoattraction of Treg cells. J Immunother Cancer. (2020) 8:e001588. doi: 10.1136/jitc-2020-001588. PMID: 33122398 PMC7597532

[23] Gong L Luo J Zhang Y Yang Y Li S Fang X . Nasopharyngeal carcinoma cells promote regulatory T cell development and suppressive activity via CD70-CD27 interaction. Nat Commun. (2023) 14:1912. doi: 10.1038/s41467-023-37614-6. PMID: 37024479 PMC10079957

[24] Lanz TV Brewer RC Ho PP Moon J-S Jude KM Fernandez D . Clonally expanded B cells in multiple sclerosis bind EBV EBNA1 and GlialCAM. Nature. (2022) 603:321–7. doi: 10.1038/s41586-022-04432-7. PMID: 35073561 PMC9382663

[25] Cencioni MT Mattoscio M Magliozzi R Bar-Or A Muraro PA . B cells in multiple sclerosis — from targeted depletion to immune reconstitution therapies. Nat Rev Neurol. (2021) 17:399–414. doi: 10.1038/s41582-021-00498-5. PMID: 34075251

[26] Soldan SS Su C Monaco MC Yoon L Kannan T Zankharia U . Multiple sclerosis patient derived spontaneous B cells have distinct EBV and host gene expression profiles in active disease. Nat Microbiol. (2024) 9:1540–54. doi: 10.1038/s41564-024-01699-6. PMID: 38806670 PMC11900839

[27] Mechelli R Umeton R Bellucci G Bigi R Rinaldi V Angelini DF . A disease-specific convergence of host and Epstein–Barr virus genetics in multiple sclerosis. Proc Natl Acad Sci USA. (2025) 122:e2418783122. doi: 10.1073/pnas.2418783122. PMID: 40184175 PMC12002260

[28] Wang H Nicholas MW Conway KL Sen P Diz R Tisch RM . EBV latent membrane protein 2A induces autoreactive B cell activation and TLR hypersensitivity. J Immunol. (2006) 177:2793–802. doi: 10.4049/jimmunol.177.5.2793. PMID: 16920914

[29] Läderach F Piteros I Fennell É Bremer E Last M Schmid S . EBV induces CNS homing of B cells attracting inflammatory T cells. Nature. (2025) 646:171–9. doi: 10.1038/s41586-025-09378-0. PMID: 40770101

[30] Helmink BA Reddy SM Gao J Zhang S Basar R Thakur R . B cells and tertiary lymphoid structures promote immunotherapy response. Nature. (2020) 577:549–55. doi: 10.1038/s41586-019-1922-8. PMID: 31942075 PMC8762581

[31] Huang Y-M Wang L-Q Liu Y Tang F-Q Zhang W-L . Integrated analysis of bulk and single-cell RNA sequencing reveals the interaction of PKP1 and tumor-infiltrating B cells and their therapeutic potential for nasopharyngeal carcinoma. Front Genet. (2022) 13:935749. doi: 10.3389/fgene.2022.935749. PMID: 36186467 PMC9515358

[32] Mrad MF Saba ES Nakib L Khoury SJ . Exosomes from subjects with multiple sclerosis express EBV-derived proteins and activate monocyte-derived macrophages. Neurol Neuroimmunol. Neuroinflamm. (2021) 8:e1004. doi: 10.1212/NXI.0000000000001004. PMID: 34006621 PMC8130999

[33] Orr N Steinman L . Epstein–Barr virus and the immune microenvironment in multiple sclerosis: insights from high-dimensional brain tissue imaging. Proc Natl Acad Sci USA. (2025) 122:e2425670122. doi: 10.1073/pnas.2425670122. PMID: 40063794 PMC11929469

[34] Chen Y Ouyang D Wang Y Pan Q Zhao J Chen H . EBV promotes TCR-T-cell therapy resistance by inducing CD163+M2 macrophage polarization and MMP9 secretion. J Immunother Cancer. (2024) 12:e008375. doi: 10.1136/jitc-2023-008375. PMID: 38886114 PMC11184188

[35] Li Y Luo C Jiang J He S Liu Y Yan W . Single‐cell analysis reveals Malignant cells reshape the cellular landscape and foster an immunosuppressive microenvironment of extranodal NK/T‐cell lymphoma. Adv Sci. (2023) 10:2303913. doi: 10.1002/advs.202303913. PMID: 37949673 PMC10754138

[36] Huang D Song S-J Wu Z-Z Wu W Cui X-Y Chen J-N . Epstein–Barr virus-induced VEGF and GM-CSF drive nasopharyngeal carcinoma metastasis via recruitment and activation of macrophages. Cancer Res. (2017) 77:3591–604. doi: 10.1158/0008-5472.CAN-16-2706. PMID: 28484077

[37] Vietzen H Berger SM Kühner LM Furlano PL Bsteh G Berger T . Ineffective control of Epstein-Barr-virus-induced autoimmunity increases the risk for multiple sclerosis. Cell. (2023) 186:5705–5718.e13. doi: 10.1016/j.cell.2023.11.015. PMID: 38091993

[38] Bickham K Goodman K Paludan C Nikiforow S Tsang ML Steinman RM . Dendritic cells initiate immune control of Epstein-Barr virus transformation of B lymphocytes *In Vitro*. J Exp Med. (2003) 198:1653–63. doi: 10.1084/jem.20030646. PMID: 14657218 PMC2194129

[39] Chen Y Sun H Liu G Wang B Wang F Sun B . EBV LMP2A-specific T cell immune responses elicited by dendritic cells loaded with LMP2A protein. Cell Mol Immunol. (2009) 6:269–76. doi: 10.1038/cmi.2009.36. PMID: 19728928 PMC4002718

[40] Li L Liu D Hutt-Fletcher L Morgan A Masucci MG Levitsky V . Epstein-Barr virus inhibits the development of dendritic cells by promoting apoptosis of their monocyte precursors in the presence of granulocyte macrophage-colony-stimulating factor and interleukin-4. Blood. (2002) 99:3725–34. doi: 10.1182/blood.v99.10.3725. PMID: 11986229

[41] Severa M Giacomini E Gafa V Anastasiadou E Rizzo F Corazzari M . EBV stimulates TLR- and autophagy-dependent pathways and impairs maturation in plasmacytoid dendritic cells: Implications for viral immune escape. Eur J Immunol. (2013) 43:147–58. doi: 10.1002/eji.201242552. PMID: 22996354

[42] Mai H-Q Chen Q-Y Chen D Hu C Yang K Wen J . Toripalimab plus chemotherapy for recurrent or metastatic nasopharyngeal carcinoma. JAMA. (2023) 330:1961–70. doi: 10.1001/jama.2023.20181. PMID: 38015220 PMC10685882

[43] Ansell SM Lesokhin AM Borrello I Halwani A Scott EC Gutierrez M . PD-1 blockade with nivolumab in relapsed or refractory Hodgkin’s lymphoma. N. Engl J Med. (2015) 372:311–9. doi: 10.1056/NEJMoa1411087. PMID: 25482239 PMC4348009

[44] Chen R Zinzani PL Fanale MA Armand P Johnson NA Brice P . Phase II study of the efficacy and safety of pembrolizumab for relapsed/refractory classic Hodgkin lymphoma. J Clin Oncol. (2017) 35:2125–32. doi: 10.1200/JCO.2016.72.1316. PMID: 28441111 PMC5791843

[45] Lim D-T Kao H-F Suteja L Li CH Quah HS Tan D-W . Clinical efficacy and biomarker analysis of dual PD-1/CTLA-4 blockade in recurrent/metastatic EBV-associated nasopharyngeal carcinoma. Nat Commun. (2023) 14:2781. doi: 10.1038/s41467-023-38407-7. PMID: 37188668 PMC10184620

[46] Montalban X Hauser SL Kappos L Arnold DL Bar-Or A Comi G . Ocrelizumab versus placebo in primary progressive multiple sclerosis. N. Engl J Med. (2017) 376:209–20. doi: 10.1056/NEJMoa1606468. PMID: 28002688

[47] Hauser SL Bar-Or A Cohen JA Comi G Correale J Coyle PK . Ofatumumab versus teriflunomide in multiple sclerosis. N. Engl J Med. (2020) 383:546–57. doi: 10.1056/NEJMoa1917246. PMID: 32757523

[48] Steinman L Fox E Hartung H-P Alvarez E Qian P Wray S . Ublituximab versus teriflunomide in relapsing multiple sclerosis. N. Engl J Med. (2022) 387:704–14. doi: 10.1056/NEJMoa2201904. PMID: 36001711

[49] Coiffier B Lepage E Brière J Herbrecht R Tilly H Bouabdallah R . CHOP chemotherapy plus rituximab compared with CHOP alone in elderly patients with diffuse large-B-cell lymphoma. N. Engl J Med. (2002) 346:235–42. doi: 10.1056/NEJMoa011795. PMID: 11807147

[50] Neelapu SS Locke FL Bartlett NL Lekakis LJ Miklos DB Jacobson CA . Axicabtagene ciloleucel CAR T-cell therapy in refractory large B-cell lymphoma. N. Engl J Med. (2017) 377:2531–44. doi: 10.1056/NEJMoa1707447. PMID: 29226797 PMC5882485

[51] Schuster SJ Bishop MR Tam CS Waller EK Borchmann P McGuirk JP . Tisagenlecleucel in adult relapsed or refractory diffuse large B-cell lymphoma. N. Engl J Med. (2019) 380:45–56. doi: 10.1056/NEJMoa1804980. PMID: 30501490

[52] Abramson JS Palomba ML Gordon LI Lunning MA Wang M Arnason J . Lisocabtagene maraleucel for patients with relapsed or refractory large B-cell lymphomas (TRANSCEND NHL 001): a multicentre seamless design study. Lancet. (2020) 396:839–52. doi: 10.1016/S0140-6736(20)31366-0. PMID: 32888407

[53] Bar-Or A Dufek M Budincevic H Drulovic J Habek M Hua LH . Safety and efficacy of fenebrutinib in relapsing multiple sclerosis (FENopta): a multicentre, double-blind, randomised, placebo-controlled, phase 2 trial and open-label extension study. Lancet Neurol. (2025) 24:656–66. doi: 10.1016/S1474-4422(25)00174-7. PMID: 40683275

[54] Wang ML Rule S Martin P Goy A Auer R Kahl BS . Targeting BTK with ibrutinib in relapsed or refractory mantle-cell lymphoma. N. Engl J Med. (2013) 369:507–16. doi: 10.1056/NEJMoa1306220. PMID: 23782157 PMC4513941

[55] Wang M Rule S Zinzani PL Goy A Casasnovas O Smith SD . Acalabrutinib in relapsed or refractory mantle cell lymphoma (ACE-LY-004): a single-arm, multicentre, phase 2 trial. Lancet Lond. Engl. (2018) 391:659–67. doi: 10.1016/S0140-6736(17)33108-2. PMID: 29241979 PMC7864374

[56] Song Y Zhou K Zou D Zhou J Hu J Yang H . Treatment of patients with relapsed or refractory mantle-cell lymphoma with zanubrutinib, a selective inhibitor of Bruton’s tyrosine kinase. Clin Cancer Res Off. J Am Assoc Cancer Res. (2020) 26:4216–24. doi: 10.1158/1078-0432.CCR-19-3703. PMID: 32461234

[57] Navarra SV Guzmán RM Gallacher AE Hall S Levy RA Jimenez RE . Efficacy and safety of belimumab in patients with active systemic lupus erythematosus: a randomised, placebo-controlled, phase 3 trial. Lancet. (2011) 377:721–31. doi: 10.1016/S0140-6736(10)61354-2. PMID: 21296403

[58] Tang X Zhou Y Li W Tang Q Chen R Zhu J . T cells expressing a LMP1-specific chimeric antigen receptor mediate antitumor effects against LMP1-positive nasopharyngeal carcinoma cells *in vitro* and *in vivo*. J BioMed Res. (2014) 28:468–75. doi: 10.7555/JBR.28.20140066. PMID: 25469116 PMC4250525

[59] Kanekiyo M Bu W Joyce MG Meng G Whittle JRR Baxa U . Rational design of an Epstein-Barr virus vaccine targeting the receptor-binding site. Cell. (2015) 162:1090–100. doi: 10.1016/j.cell.2015.07.043. PMID: 26279189 PMC4757492

[60] Taylor GS Jia H Harrington K Lee LW Turner J Ladell K . A recombinant modified vaccinia ankara vaccine encoding Epstein-Barr virus (EBV) target antigens: a phase I trial in UK patients with EBV-positive cancer. Clin Cancer Res Off. J Am Assoc Cancer Res. (2014) 20:5009–22. doi: 10.1158/1078-0432.CCR-14-1122-T. PMID: 25124688 PMC4340506

[61] Peng X He X Zhang W Huang H Li X Chen J . Safety, tolerability, and immunogenicity of WGc-043 in subjects with EBV-positive cancers: Results from an investigator-initiated trial. J Clin Oncol. (2024) 42:139. doi: 10.1200/JCO.2024.42.23_suppl.139

[62] Li C Wang D Hasegawa A Liu H An N Bao Y . Ksd-101 in patients with EBV-associated hematologic neoplasms: results from an ongoing phase I clinical study. Blood. (2023) 142:4837. doi: 10.1182/blood-2023-182556

[63] Wu M Hau PM Li L Tsang CM Yang Y Taghbalout A . Synthetic BZLF1-targeted transcriptional activator for efficient lytic induction therapy against EBV-associated epithelial cancers. Nat Commun. (2024) 15:3729. doi: 10.1038/s41467-024-48031-8. PMID: 38702330 PMC11068728

[64] Sabatino JJ Cree BAC Hauser SL . New horizons for multiple sclerosis therapy: 2025 and beyond. Ann Neurol. (2025) 98:317–28. doi: 10.1002/ana.27270. PMID: 40474602 PMC12278195

[65] Montalban X Vermersch P Arnold DL Bar-Or A Cree BAC Cross AH . Safety and efficacy of evobrutinib in relapsing multiple sclerosis (evolutionRMS1 and evolutionRMS2): two multicentre, randomised, double-blind, active-controlled, phase 3 trials. Lancet Neurol. (2024) 23:1119–32. doi: 10.1016/S1474-4422(24)00328-4. PMID: 39307151

[66] Fox RJ Bar-Or A Traboulsee A Oreja-Guevara C Giovannoni G Vermersch P . Tolebrutinib in nonrelapsing secondary progressive multiple sclerosis. N. Engl J Med. (2025) 392:1883–92. doi: 10.1056/NEJMoa2415988. PMID: 40202696

[67] Oh J Arnold DL Cree BAC Ionete C Kim HJ Sormani MP . Tolebrutinib versus teriflunomide in relapsing multiple sclerosis. N. Engl J Med. (2025) 392:1893–904. doi: 10.1056/NEJMoa2415985. PMID: 40202623

[68] Saegusa K Tsuchida Y Komai T Tsuchiya H Fujio K . Advances in targeted therapy for systemic lupus erythematosus: Current treatments and novel approaches. Int J Mol Sci. (2025) 26:929. doi: 10.3390/ijms26030929. PMID: 39940698 PMC11816971

[69] Navarra SV Guzmán RM Gallacher AE Hall S Levy RA Jimenez RE . Efficacy and safety of belimumab in patients with active systemic lupus erythematosus: a randomised, placebo-controlled, phase 3 trial. Lancet Lond. Engl. (2011) 377:721–31. doi: 10.1016/S0140-6736(10)61354-2. PMID: 21296403

[70] Yoshizaki T Kondo S Endo K Nakanishi Y Aga M Kobayashi E . Modulation of the tumor microenvironment by Epstein‐Barr virus latent membrane protein 1 in nasopharyngeal carcinoma. Cancer Sci. (2018) 109:272–8. doi: 10.1111/cas.13473. PMID: 29247573 PMC5797826

[71] Liu Y He S Wang X-L Peng W Chen Q-Y Chi D-M . Tumour heterogeneity and intercellular networks of nasopharyngeal carcinoma at single cell resolution. Nat Commun. (2021) 12:741. doi: 10.1038/s41467-021-21043-4. PMID: 33531485 PMC7854640

[72] Xu X Zhu N Zheng J Peng Y Zeng M-S Deng K . EBV abortive lytic cycle promotes nasopharyngeal carcinoma progression through recruiting monocytes and regulating their directed differentiation. PloS Pathog. (2024) 20:e1011934. doi: 10.1371/journal.ppat.1011934. PMID: 38206974 PMC10846743

[73] Cao Y Xie L Shi F Tang M Li Y Hu J . Targeting the signaling in Epstein–Barr virus-associated diseases: mechanism, regulation, and clinical study. Signal Transd. Targ. Ther. (2021) 6:15. doi: 10.1038/s41392-020-00376-4. PMID: 33436584 PMC7801793

[74] Yap LF Wong AKC Paterson IC Young LS . Functional implications of Epstein-Barr virus lytic genes in carcinogenesis. Cancers. (2022) 14:5780. doi: 10.3390/cancers14235780. PMID: 36497262 PMC9740547

